# Fracture Detection on Bone Radiographs: The Impact of an AI Tool on Orthopaedic Night Shifts

**DOI:** 10.3390/jimaging12060252

**Published:** 2026-06-07

**Authors:** Domenico Albano, Giacomo Vignati, Sara D’Andrea, Salvatore Gitto, Carmelo Messina, Riccardo Accetta, Luca Maria Sconfienza

**Affiliations:** 1 Department of Radiology, ASST Grande Ospedale Metropolitano Niguarda, Piazza dell’Ospedale Maggiore, 3, 20162 Milano, Italy; 2 Dipartimento di Scienze Biomediche, Chirurgiche ed Odontoiatriche, Università Degli Studi di Milano, Via della Commenda 10, 20122 Milano, Italy; 3 ASST Valle Olona, Busto Arsizio, 21052 Varese, Italy; giacomovignati95@gmail.com; 4 School of Radiology, Università Degli Studi di Milano, Via Festa del Perdono, 7, 20122 Milano, Italy; sara.dandrea@unimi.it; 5 Department of Biomedical Sciences for Health, Università Degli Studi di Milano, Via Festa del Perdono, 7, 20122 Milano, Italy; 6 IRCCS Istituto Ortopedico Galeazzi, Via Cristina Belgioioso 173, 20157 Milano, Italy; 7 UOC Radiodiagnostica, ASST Centro Specialistico Ortopedico Traumatologico Gaetano Pini-CTO, P.zza Cardinal Ferrari, 1, 20122 Milano, Italy

**Keywords:** fracture, detection, radiograph, artificial intelligence, trauma, diagnostic accuracy, night shift, orthopaedist

## Abstract

We evaluated how using an artificial intelligence (AI)-based diagnostic tool impacts orthopaedists’ accuracy in detecting fractures during night shifts without the support of on-site radiologists. We compared diagnostic discrepancies between orthopaedists and radiologists in recorded cases from our emergency department between September 2024 and June 2025. In February 2025, we introduced Gleamer BoneView^®^ 2.6.0 to help orthopaedists with automated fracture detection during shifts without on-site radiologists. Statistical analyses measured the rates of fracture misdiagnosis before and after implementation of Gleamer BoneView^®^. Chi-square and Fisher’s Exact Tests were employed, and a *p*-value < 0.05 was considered statistically significant. A total of 28,655 patients were subjected to radiographs resulting in 31/13,813 recalls (0.22%) in the pre-implementation and 27/14,842 recalls in the post-implementation period (0.18%, *p* = 0.42). Among these, 51 recalls (30 males, age: 39 ± 23 years) were related to fractures: 26 (16 missed fractures, 8 clinical re-assessment, 2 additional CT) occurred in the pre-implementation period (0.19%), and 25 (13 missed fractures, 7 clinical reassessment, 3 additional radiographs, 2 additional CT) in the post-implementation period (0.17%, *p* = 0.63). Patient management was changed in 9/13,813 patients (0.0006%) in the pre-implementation period and 7/14,842 (0.0005%) after the implementation (*p* = 0.551). Gleamer BoneView^®^ use was linked to a non-significant decrease in missed fractures by orthopaedists working without on-site radiologists.

## 1. Introduction

Bone fractures are common; about 30–50% of men and 40–60% of women will face at least one in their lifetime. These fractures can cause long-term disability, loss of work ability, and lower productivity. This creates a significant economic burden on patients, families, and healthcare systems. Today, the global rate of fractures is rising, largely due to road traffic accidents and existing bone issues, such as osteoporosis in older patients and metastatic diseases in cancer patients [1,2].

Conventional radiography (CR) remains the primary imaging modality for the initial evaluation of suspected fractures, especially in the emergency department (ED), due to its low cost, relatively low radiation exposure, and broad availability [2]. The standard clinical workflow in the ED consists of taking the patient’s medical history, especially details of how the injury occurred, followed by physical examination. If a fracture is suspected, the clinician will usually request a CR of the affected anatomical region [3]. Although additional radiographic projections can be taken, computed tomography is used when necessary. However, CR has inherent limitations, including low sensitivity for detecting certain fractures and interobserver variability. It is estimated that 1–3.7% of fractures may be missed during the initial evaluation, particularly in specific anatomical areas (e.g., scapula, scaphoid, calcaneus, ribs, thoracic spine) [4,5]. An increased workload, an insufficient clinical history, and a shortage of trained radiologists all contribute to an increased risk of error. Additionally, radiologists are prone to common diagnostic errors, such as becoming satisfied with initial search results, and may therefore miss multiple fractures [6,7]. Wei et al., in a study of extremity fractures presenting to the ED, identified 115 missed fractures among 3081 cases, of which only one-third were considered radiographically undetectable [8]. Other studies have reported missed fracture rates of up to 3% at the initial evaluation, of which 86% subsequently required treatment modifications [5,9,10,11,12,13].

In recent years, several commercially available artificial intelligence (AI) systems have been developed to identify fractures, with the aim of supporting radiologists and emergency physicians in routine clinical practice, not only by detecting subtle features that are challenging for the human eye, but also by mitigating cognitive errors from fatigue or satisfaction bias during image interpretation [9,10,13,14,15]. Among these, Gleamer BoneView^®^ (Gleamer, Paris, France) is a deep learning software that has demonstrated particularly promising performance in fracture detection on CRs, achieving an area under the curve (AUC) of up to 0.97 in independent assessments [11,12]. Furthermore, additional studies proved an overall increase in sensitivity (8.7%) and specificity (4.1%) for adult appendicular skeletal fractures [16]; notably, sensitivity was significantly higher (*p* < 0.05) among the vast majority of readers when evaluating CR with Gleamer BoneView^®^, all without reducing reading speed [17]. All images from a CR are automatically elaborated, and within less than three minutes, the system returns a preliminary diagnosis classified as positive, doubtful, or negative. In a retrospective case–control investigation including 600 patients, the use of the Gleamer BoneView^®^ algorithm was associated with improved diagnostic performance [18]. Specifically, AI support increased readers’ sensitivity for fracture detection by 8.7% and specificity by 4.1%. In some institutions, there are no radiologists on duty or on call; therefore, CRs are assessed by the orthopaedists on duty, with non-negligible discrepancies between orthopaedists and radiologists in evaluating CR exams of patients admitted to the ED [19,20]. The diagnostic challenges increase during night shifts, particularly when orthopaedists work without an on-site radiologist, with the burden of imaging interpretation being completely upon the attending orthopaedist, thereby increasing the risk of diagnostic discrepancies and wrong or delayed treatments. Also, this workflow introduces further specific challenges in conditions of sleep deprivation and cognitive fatigue. To our knowledge, a literature gap exists concerning how AI-based solutions, such as BoneView^®^ (Gleamer), may be helpful for orthopaedists in imaging interpretation in such stressful conditions.

This pilot study aims to evaluate how using an AI-based diagnostic tool, namely Gleamer BoneView^®^ (https://www.gleamer.ai/copilot/boneview), impacts orthopaedists’ accuracy in detecting fractures in traumas during night shifts in the ED without the support of an on-site radiologist.

## 2. Materials and Methods

### 2.1. Study Design

This retrospective study was approved by the local Ethics Committee (Comitato Etico Territoriale Lombardia 1, Protocol RETRORAD, approved on 12 January 2026), and the need for informed consent was waived. Following the matching of imaging, laboratory, and surgical data, the database was fully anonymised to eliminate any links between the data and patients’ identities, in compliance with the General Data Protection Regulation for Research Hospitals.

This study was conducted at IRCCS Ospedale Galeazzi-Sant’Ambrogio, a tertiary orthopaedic hospital specialised in orthopaedic care, with an ED focused on minor trauma, while polytrauma cases are directed to a nearby general hospital. Our ED is staffed by a board-certified orthopaedist with 5–30 years of experience in trauma care, supported by residents, 24 h per day. In addition, a certified radiologist with 5–15 years of expertise in musculoskeletal imaging is present for 12 h per day. During night shifts, radiology coverage is not available, so CRs are interpreted by the attending orthopaedist in accordance with institutional policy. All imaging studies are subsequently archived and formally reviewed the following morning by the attending radiologist. Whenever the radiologist’s interpretation differs from the initial orthopaedic assessment, a discrepancy form is completed in collaboration with the orthopaedist on duty, and the patient is recorded in a dedicated discrepancy registry. This process enables patient recall for repeat clinical evaluation, additional imaging when indicated, and modification of management if required.

**Figure 1:**
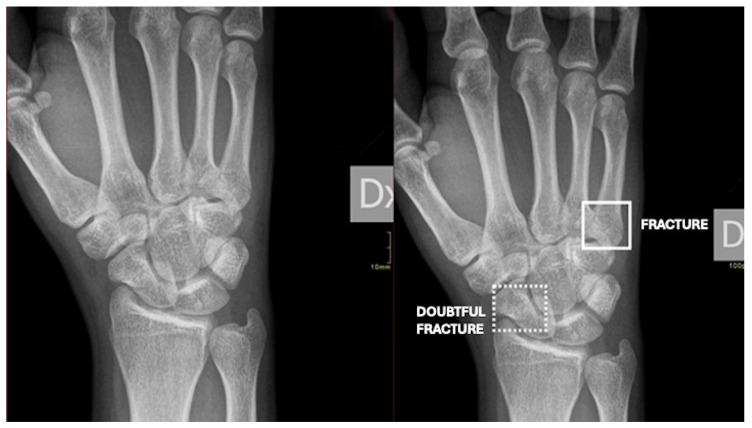
Right-wrist CR with Gleamer BoneView^®^ indicating a fracture of the fifth metacarpal bone and a doubtful fracture of the scaphoid bone.

**Figure 2:**
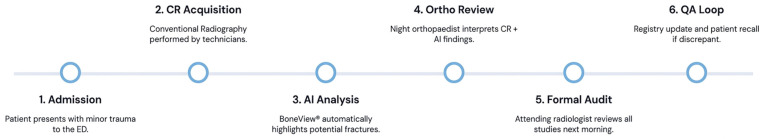
Shows the complete workflow, from patient presentation and image acquisition to AI-assisted analysis, orthopaedist interpretation, and next-day radiologist review.

We retrospectively included in our study all cases of minor trauma in the ED discrepancy register over 10 months (September 2024–June 2025) where diagnostic errors occurred without radiologist coverage. Gleamer BoneView^®^ was implemented from February 2025 to June 2025 to support orthopaedists in the ED. In this period, after clinical examination by the attending orthopaedist, the patient has been subjected to CRs. After a few minutes, the CR images were available in the PACS System together with the results of Gleamer BoneView^®^ analysis automatically highlighting fractures. Gleamer BoneView^®^ classifies lesions into Negative, Doubtful (dotted box), or Positive (solid box), with the box placed in the CR image to indicate the area of injury (Figure 1). Hence, the orthopaedist could evaluate the patient based on CR images and Gleamer BoneView^®^ results, then provide the optimal treatment. Further, AI overlays were also made visible to the attending radiologist the next morning (Figure 2).

### 2.2. Data and Statistical Analysis

All collected data regarding diagnostic discrepancies and patient recalls after CRs were extracted from the ED discrepancy register, which was reviewed by a radiology resident with five years of experience in musculoskeletal imaging. The radiology resident was blinded to the pre/post-implementation period since he did not know the study design at that time. Moreover, the introduction of AI was not reported in the discrepancy register so that he was blind to the impact of the AI tool on data. We compared discrepancies between orthopaedic and radiologist evaluations in two intervals: pre-implementation of Gleamer BoneView^®^ (September 2024–January 2025) and post-implementation (February–June 2025). This included overall and region-specific changes in misdiagnosed-injury incidence, with statistical significance assessed. Misdiagnosed fractures were initially classified by specific anatomical sites and subsequently grouped into three major anatomical regions: (i) upper limb; (ii) lower limb; and (iii) axial skeleton (sternum, ribs, spine). The relative incidence of misdiagnosed injuries within each major region and anatomical site was calculated as a percentage of the total recalls. Injuries were further categorised according to the cause of recall: (i) missed injury detected by the radiologist in a patient considered to have no injuries by the orthopaedist; (ii) orthopaedic clinical reassessment for discrepancies between orthopaedist and radiologist in injuries interpretation (e.g., more fractures than those initially detected, displaced fractures initially considered undisplaced); (iii) additional projections; and (iv) further imaging (e.g., CT, MRI).

Comparisons were conducted between the pre- and post-implementation periods to assess changes in the number of recalls and misdiagnosed injuries. Descriptive statistics were used, and results are reported as means ± standard deviation and percentages. For the overall dataset and for analyses at the level of major anatomical regions, differences were evaluated using the Chi-square test. In cases involving individual anatomical sites, including those with zero events, Fisher’s Exact Test was employed to ensure accurate estimation of statistical significance. A *p*-value of less than 0.05 was considered indicative of statistical significance.

## 3. Results

Between September 2024 and January 2025, a total of 13,813 patients were subjected to CRs due to trauma during night shifts in the ED, resulting in 31 recalls (0.22%). Between February and June 2025, CRs were performed in 14,842 patients due to trauma, with 27 recalls (0.18%). Although a slight reduction in recalls was observed after the introduction of Gleamer Bone View^®^, the difference was not statistically significant (*p* = 0.42). Of these 58 recalls, 7 cases (5 cases in the pre-implementation period and 2 cases in the post-implementation period) were traumas without any fractures.

The 51 recalls (30 males, 21 females, mean age: 39 ± 23 years, range 8–83 years) for traumas with fractures were categorised as follows:

- A total of 26 patients (17 males, 9 females, mean age: 40 ± 29 years, range 8–83 years) with fractures in the pre-implementation period (0.19%) and 25 patients (13 males, 12 females, mean age: 40 ± 29 years, range 10–81 years) with fractures in the post-implementation period (0.17%, *p* = 0.63).
- Missed fractures were the most common cause of trauma-related recall in both periods. In the pre-implementation period, 16 patients with missed fractures were identified compared with 13 patients in the post-implementation period (*p* = 0.46).
- The number of recalls due to fractures that required clinical reassessment by the orthopaedist after review by the attending radiologist was eight patients in the pre-implementation period and seven patients in the post-implementation period (*p* = 0.88).
- Concerning additional imaging performed after recall, three patients required additional CR projections in the post-implementation period, whereas no cases required additional CR in the pre-implementation period (*p* = 0.11). Additional CT scans were requested in two cases in both periods (*p* = 1.00) (Table 1).

The most frequently affected regions were the foot (n = 10/51 patients, 19.6%), forearm (n = 8/51 patients, 15.7%), and ankle and knee (n = 4/51 patients each, 7.8%). Other sites, including femur, leg, hip–pelvis, clavicle, shoulder, upper arm, elbow, wrist, and axial skeleton (sternum, ribs, and spine), were less commonly involved. All the discrepancies were stratified by three major anatomical regions: upper limb (n = 23/51 patients, 45.1%), lower limb (n = 22/51 patients, 43.1%), and axial skeleton (n = 6/51 patients, 11.8%). The difference in the distribution of misdiagnosed fractures across the three major regions was statistically significant (*p* < 0.01), with the upper and lower limbs being more frequently affected than the axial skeleton.

**Figure 3:**
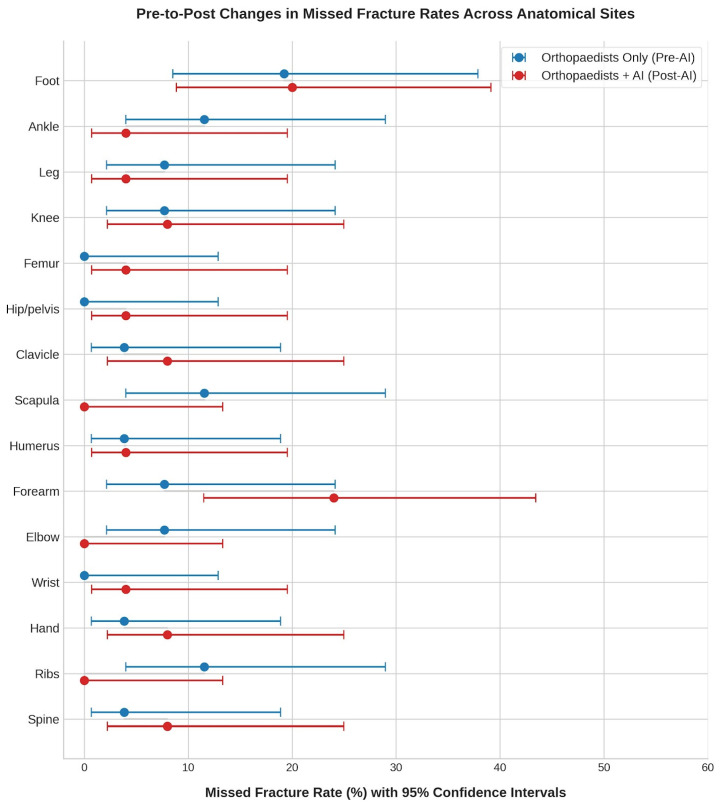
Forest plot to visually present the pre-to-post changes in missed fracture rates across anatomical sites with confidence intervals.

After the introduction of AI in February 2025, the anatomical sites showing the greatest reduction in diagnostic errors were the scapula and ribs (both from 3/26 to 0/25 recalls, *p* = 0.11). When comparing the three major anatomical regions (upper limb, lower limb, axial skeleton), no statistically significant differences were observed between the two periods (*p* > 0.36). There were no significant reductions at specific anatomical sites (*p* ≥ 0.11). Table 2 and Figure 3 show the anatomic distributions of fractures.

Upon reviewing our discrepancy registry, we observed that patient management changed in 16 cases: 9 out of 13,813 patients (0.0006%) in the pre-implementation period and 7 out of 14,842 (0.0005%) in the post-implementation period (*p* = 0.551). All instances involved missed fractures that required a different, yet strictly conservative, treatment strategy; surgical intervention was never indicated for cases where it had not been initially planned. The affected anatomical sites included fractures of the hand (n = 1 pre-AI, n = 1 post-AI), pelvis (n = 0 pre-AI, n = 1 post-AI), ankle (n = 1 pre-AI, n = 0 post-AI), spine (n = 1 pre-AI, n = 1 post-AI), foot (n = 2 pre-AI, n = 1 post-AI), scapula (n = 2 pre-AI, n = 0 post-AI), forearm (n = 1 pre-AI, n = 3 post-AI), and humerus (n = 1 pre-AI, n = 0 post-AI).

## 4. Discussion

In our study, we observed a slight reduction in recalls following the implementation of Gleamer BoneView^®^; however, this decrease was not statistically significant. The anatomical distribution of diagnostic errors revealed that the most frequently affected regions were the foot (19.6%), forearm (15.7%), ankle and knee (7.8% each), although no statistically significant differences were observed. Consistent with previous studies, no specific anatomical region appeared particularly prone to misdiagnosis [21].

Diagnostic discrepancies were further stratified into three major anatomical regions: upper limb (45.1%), lower limb (43.1%), and axial skeleton (11.8%). The distribution of errors across these regions was statistically significant (*p* < 0.01), with the upper and lower limbs more commonly affected than the axial skeleton. These findings align with the current literature, where AI-based fracture detection has shown the highest performance for extremity fractures, including the wrist, hand, shoulder, ankle and foot [22]. It is important to consider that trauma CRs are more frequently performed on the extremities than on the axial skeleton. Studies indicate that the number of emergency trauma CRS performed on the extremities (upper and lower limbs) is substantially higher than on the axial skeleton.

Over a ten-month period, evaluation of Gleamer BoneView^®^ showed a non-significant reduction in diagnostic errors [20]. Overall, the implementation of Gleamer BoneView was associated with a non-significant decrease in recalls (*p* = 0.36). Prior studies demonstrated AI’s potential to enhance fracture detection sensitivity. For instance, a study at the Trauma and Orthopaedic Hospital in Riga reported a significant reduction in diagnostic errors, with accuracy improving from 78.1% to 85.2% following AI assistance [13]. It also highlights the effectiveness of AI tools such as Gleamer BoneView^®^ in supporting more experienced physicians under conditions of physical fatigue, as well as assisting less experienced physicians in their learning process. Another recent study by Dell’Aria [23] compared the effectiveness of fracture recognition between an AI tool, a senior radiologist, and a junior radiology resident. The study demonstrated that the AI outperformed the junior resident and performed comparably to the senior radiologist in detecting common fractures, while its accuracy remained lower than that of the senior radiologist in identifying more subtle fractures.

Previous studies have not investigated the impact of this AI tool on orthopaedists’ diagnostic performance in fracture recognition in an emergency setting without radiologists on-site. Although AI may serve as a valuable tool to support physicians, enhance fracture management, and improve diagnostic accuracy, it seems that it does not substantially impact the management of minor traumas managed by skilled orthopaedists when radiologist availability is limited. Indeed, patient management changed in just 0.0005–0.0006% of the cases. All instances involved missed fractures that required a different, yet strictly conservative, treatment strategy. Our results may be explained by the low number of recalls that limit the statistical power of our data. Further, the high expertise of orthopaedists who have been working in a highly specialised referral orthopaedic centre, which is also a university site for the postgraduate school of orthopaedics, may have improved their confidence and accuracy in CR evaluation, as proven by the quite low rate of discrepancies with radiologists’ evaluation. The notion that extensive experience and highly specialised training may have influenced our results is a hypothesis we propose to partially explain the data. This effect, however, stems less from the total years of experience than from the specific environment in which our orthopaedists were trained and practice daily: a tertiary orthopaedic hospital where the specialists rotating through the ED are highly specialised in traumatology. Therefore, this study underscores the value of interpreting CR images and AI-derived results in view of clinical data by a physician who deals with patients and not just with images.

On the other hand, it should be considered that the very low missed-fracture rate makes it statistically complicated to demonstrate an incremental benefit from AI integration in this clinical scenario within our sample size [24]. In low-event-rate settings, standard statistical power may fall short of identifying subtle improvements [25]. While the benefit in terms of diagnostic performance and patient management seems to be limited in our series, greater improvements might be observed in specific subgroups, like less experienced orthopaedists, specific fractures or anatomical regions. Larger multi-centre works focused on specific subgroup analyses should investigate whether AI tools may reach targeted benefits that can be hidden in aggregate data, something often observed when exploring AI tools in settings with already high clinical accuracy.

Notably, AI overlays were visible to the radiologist, creating circularity in the post-implementation period and somewhat affecting the radiologist’s interpretation, which is the gold standard of this study. We believe that this does not weaken the study’s validity. Indeed, a recent meta-analysis on the diagnostic performance of commercially available AI solutions for fracture detection on CRs has reported a significant increase in pooled sensitivity of unaided human readers (0.67) when compared with AI-aided human readers (0.80, *p* ≤ 0.001), without significant change in specificity (0.96 vs. 0.95, respectively, *p* = 0.316) [26].

Previous papers on this AI tool have shown some limitations, such as its reduced diagnostic accuracy in identifying subtle, non-displaced obvious fractures, specific avulsion injuries or complex fractures (i.e., toddler’s, buckle, or bow fractures), leading to a higher rate of false negatives for specific injury types [14,15,27,28]. Despite the huge number of patients analysed in our study, the number of discrepancies and the impact of the AI tool were probably so low that it was not possible to obtain this result in our work.

Notably, the need for further examinations in our study was related to doubtful imaging findings reported by the radiologist, who also viewed what AI highlighted in CRs. In these cases, suspicious fractures were highlighted by the AI tool and additional CR views were requested after discussion between the radiologist and orthopaedist.

Some limitations of this study should be considered. Firstly, the limited number of discrepancy cases restricts the statistical power of the study, underpowering our subgroup analysis. Its retrospective design carries an inherent risk of incomplete data collection and potential selection bias. Then, as the study was conducted at a single tertiary-level orthopaedic referral centre, the findings may not be generalisable to other hospitals or EDs with different organisational models. The study population primarily consisted of patients with minor trauma, as polytrauma cases were systematically referred to another hospital. This restricts the applicability of the results to more complex emergency scenarios. Last, we did not perform a sample size calculation since we had the AI tool available for just five months and we could not include more patients. Even performing a post hoc power analysis to assess the sample size that would have been needed to reach standard statistical power, we acknowledge that the study is basically underpowered to detect an effect at our event rate, making it a pilot study.

## 5. Conclusions

The distribution of unrecognised fractures was first assessed, revealing a higher prevalence in the appendicular skeleton compared to the axial skeleton. The implementation of Gleamer BoneView^®^ was not associated with a statistically significant reduction in the incidence of missed injuries by orthopaedists in an ED without on-site radiologist support. It is still unclear whether our results indicate that AI tools for fracture detection have a limited role for orthopaedists, or whether these findings highlight how the impact depends on the expertise of the physician and the low rate of diagnostic errors. At any rate, these results do not provide evidence to support the implementation of this AI tool in orthopaedic activity during night shifts. Further studies on larger populations are needed to understand AI performance across different institutional settings, stratifying series by physician experience and specific anatomical sub-regions to better define human–AI collaboration.

## Figures and Tables

**Table 1:** Comparison of the different causes of recalls for fracture between the pre-implementation and post-implementation period.

	Orthopaedists Only	Orthopaedists + AI	*p*-Values
Number of recalled patients for fractures	26/13,813 (0.19%)	25/14,842 (0.17%)	0.63
Missed fractures	16/26 (62%)	13/25 (52%)	0.46
Fractures requiring clinical reassessment	8/26 (31%)	7/25 (28%)	0.88
Additional CRs	0/26 (0%)	3/25 (12%)	0.11
Additional CTs	2/26 (8%)	2/25 (8%)	1.00

Note: *p* = *p*-values of Fisher’s Exact Test.

**Table 2:** Anatomic distribution of fractures in pre- and post-implementation periods.

	Orthopaedists Only	Orthopaedists + AI	*p*-Values
foot	5/26 (19%)	5/25 (20%)	1.00
ankle	3/26 (12%)	1/25 (4%)	0.34
leg	2/26 (8%)	1/25 (4%)	0.60
knee	2/26 (8%)	2/25 (8%)	1.00
femur	0/26 (0%)	1/25 (4%)	0.49
hip/pelvis	0/26 (0%)	1/25 (4%)	0.49
clavicle	1/26 (4%)	2/25 (8%)	0.60
scapula	3/26 (12%)	0/25 (0%)	0.11
humerus	1/26 (4%)	1/25 (4%)	1.00
forearm	2/26 (8%)	6/25 (24%)	0.12
elbow	2/26 (8%)	0/25 (0%)	0.24
wrist	0/26 (0%)	1/25 (4%)	0.49
hand	1/26 (4%)	2/25 (8%)	0.60
ribs	3/26 (12%)	0/25 (0%)	0.11
spine	1/26 (4%)	2/25 (8%)	0.60

Note: *p* = *p*-values of the Fisher’s Exact Test.

## Data Availability

The datasets analysed during the current study are available from the corresponding author upon reasonable request. Access to data will be provided in compliance with applicable ethical guidelines and institutional regulations.

## Abbreviations

The following abbreviations are used in this manuscript:

AI	Artificial Intelligence
AUC	Area Under the Curve
CR	Conventional Radiography
CT	Computed Tomography
ED	Emergency Department
MRI	Magnetic Resonance Imaging
PACS	Picture Archiving and Communication System

## References

[1] GBD 2019 Fracture Collaborators (2021). Global, regional, and national burden of bone fractures in 204 countries and territories, 1990–2019: A systematic analysis from the Global Burden of Disease Study 2019. Lancet Healthy Longev..

[2] Ruitenbeek H.C., Sahil S., Kumar A., Kushawaha R.K., Tanamala S., Sathyamurthy S., Agrawal R., Chattoraj S., Paramasamy J., Bos D. (2025). Cross-validation of an artificial intelligence tool for fracture classification and localization on conventional radiography in Dutch population. Insights Imaging.

[3] Oppenheimer J., Lüken S., Hamm B., Niehues S.M. (2023). A prospective approach to integration of AI fracture detection software in radiographs into clinical workflow. Life.

[4] Gitto S., Omoumi P., Albano D., Xiberta P., Rossi S., Rizzo A., Messina C., Splendiani A., Barile A., Sconfienza L.M. (2026). Artificial Intelligence in Spine Imaging Interpretation. Semin. Musculoskelet. Radiol..

[5] Omar M., Elsamaloty M., Yu D., Khan I., Shah Z., Matthew B.P., Bukhari S.M.A., Nadeem D., Gupta A. (2026). Artificial intelligence as a simultaneous second reader in diagnostic radiology: An umbrella review of systematic reviews and meta-analyses. Curr. Probl. Diagn. Radiol..

[6] Guermazi A., Tannoury C., Kompel A.J., Murakami A.M., Ducarouge A., Gillibert A., Li X., Tournier A., Lahoud Y., Jarraya M. (2022). Improving radiographic fracture recognition performance and efficiency using artificial intelligence. Radiology.

[7] Kuo R.Y.L., Harrison C., Curran T.-A., Jones B., Freethy A., Cussons D., Stewart M., Collins G.S., Furniss D. (2022). Artificial intelligence in fracture detection: A systematic review and meta-analysis. Radiology.

[8] Wei C.-J., Tsai W.-C., Tiu C.-M., Wu H.-T., Chiou H.-J., Chang C.-Y. (2006). Systematic analysis of missed extremity fractures in emergency radiology. Acta Radiol..

[9] Pervez A., Hasan S.U., Norrish A.R. (2026). Convolutional neural networks in paediatric fracture detection: Pooled evidence from a systematic review and meta-analysis. Eur. Radiol..

[10] Bruun F.J., Müller F.C., Nybing J.U., Hansen P., Gosvig K.K., Boesen M.P., Brejnebøl M.W. (2026). Independent bone-level diagnostic accuracy study of an AI tool for detecting appendicular skeletal fractures on radiographs. Eur. Radiol..

[11] Tordjman M., Fritz J., Regnard N.-E., Kijowski R., Mihoubi F., Taouli B., Mei X., Huang M., Guermazi A. (2024). Artificial intelligence in musculoskeletal radiology: Practical aspects and latest perspectives. BJR|Open.

[12] Hernáiz Ferrer A.I., Bortolotto C., Carone L., Preda E.M., Fichera C., Lionetti A., Gambini G., Fresi E., Grassi F.A., Preda L. (2025). Application of artificial intelligence in the diagnosis of scaphoid fractures: Impact of automated detection of scaphoid fractures in a real-life study. Radiol. Med..

[13] Altmann-Schneider I., Kellenberger C.J., Pistorius S.-M., Saladin C., Schäfer D., Arslan N., Fischer H.L., Seiler M. (2024). Artificial intelligence-based detection of paediatric appendicular skeletal fractures: Performance and limitations for common fracture types and locations. Pediatr. Radiol..

[14] Luiken I., Lemke T., Komenda A., Marka A.W., Kim S.H., Graf M.M., Ziegelmayer S., Weller D., Mertens C., Bressem K. (2025). Evaluation of commercial AI algorithms for the detection of fractures, effusions, and dislocations on real-world clinical data: A prospective registry study. Radiography.

[15] Boginskis V., Zadoroznijs S., Cernavska I., Beikmane D., Sauka J. (2023). Artificial intelligence effectivity in fracture detection. Medicni Perspekt..

[16] Huhtanen J.T., Nyman M., Blanco Sequeiros R., Koskinen S.K., Pudas T.K., Kajander S., Niemi P., Aronen H.J., Hirvonen J. (2025). Comparative accuracy of two commercial AI algorithms for musculoskeletal trauma detection in emergency radiographs. Emerg. Radiol..

[17] Kwee R.M., Kwee T.C. (2025). Artificial intelligence-assisted detection of fractures on radiographs with BoneView: A systematic review. Eur. J. Radiol..

[18] Duron L., Ducarouge A., Gillibert A., Lainé J., Allouche C., Cherel N., Zhang Z., Nitche N., Lacave E., Pourchot A. (2021). Assessment of an AI aid in detection of adult appendicular skeletal fractures by emergency physicians and radiologists: A multicenter cross-sectional diagnostic study. Radiology.

[19] Albano G.D., Argo A., Zerbo S., Scavone C., Vitale F., Messina C., Gitto S., Albano S., Midiri M., Vitali P. (2025). Imaging of musculoskeletal injury: Timing estimation and medico-legal issues. Radiol. Med..

[20] Catapano M., Albano D., Pozzi G., Accetta R., Memoria S., Pregliasco F., Messina C., Sconfienza L.M. (2017). Differences between orthopaedic evaluation and radiological reports of conventional radiographs in patients with minor trauma admitted to the emergency department. Injury.

[21] Hallas P., Ellingsen T. (2006). Errors in fracture diagnoses in the emergency department—Characteristics of patients and diurnal variation. BMC Emerg. Med..

[22] Elbahi M.K., Muhammed A., Fadlelmola Abdalla Mohamednour M., Mukhtar F.S. (2025). Artificial intelligence in fracture diagnosis on radiographs: Evidence, pitfalls, and pathways for clinical integration (2020–2025). Cureus.

[23] Dell’Aria A., Tack D., Saddiki N., Makdoud S., Alexiou J., De Hemptinne F.-X., Berkenbaum I., Neugroschl C., Tacelli N. (2024). Radiographic detection of post-traumatic bone fractures: Contribution of artificial intelligence software to the analysis of senior and junior radiologists. J. Belg. Soc. Radiol..

[24] Kumar P., Alnaimi N.A., Soman S., Suansing L., Ryan Arriola D., Jamea L.A. (2026). Meta-Analysis on Comparison of Diagnostic Accuracy Between Artificial Intelligence and Healthcare Professionals. Sci.

[25] Storey M., Chung A., Packer J., Bartsch A.M., Rhodes A., Colta R., Rickaby S., Malamateniou C., Dean G., Shelmerdine S. (2026). AI Triage of Normal Chest Radiographs: A Silent Trial and Failure Analysis. Radiol. Artif. Intell..

[26] Husarek J., Hess S., Razaeian S., Ruder T.D., Sehmisch S., Müller M., Liodakis E. (2024). Artificial intelligence in commercial fracture detection products: A systematic review and meta-analysis of diagnostic test accuracy. Sci. Rep..

[27] Kanesan H., Choudhary Z., Singal S., Kanesan M., Hang-Kin Nam R., Radhamony N.G., Hamadto M. (2025). Missed on X-ray, Found on CT: A Retrospective Study on the Diagnostic Yield and Clinical Consequences of Occult Posterior Malleolus Fractures in Tibial Shaft Fractures. Cureus.

[28] Albano D., Basile M., Fusco S., Asmundo L., Gitto S., Messina C., Piacentini A., Rizzetto F., Monti C.B., Zanardo M. (2026). AI in Musculoskeletal Imaging: An End-to-End Perspective. J. Clin. Med..

